# Assessment of the Novel Anti-Seizure Potential of Validamycin A Using Zebrafish Epilepsy Model

**DOI:** 10.3390/molecules29112572

**Published:** 2024-05-30

**Authors:** Eunhye Lee, Amit Banik, Ki-Baek Lee, Seung Min Sim, Ah Hyun Kil, Byung Joon Hwang, Yun Kee

**Affiliations:** 1 Department of Biomedical Science, College of Biomedical Science, Kangwon National University, Chuncheon 24341, Republic of Korea; eunhye78@kangwon.ac.kr (E.L.); losa625@kangwon.ac.kr (S.M.S.); 2 Interdisciplinary Graduate Program in Environmental and Biomedical Convergence, College of Biomedical Science, Kangwon National University, Chuncheon 24341, Republic of Korea; natureamitbanik@kangwon.ac.kr; 3 Zefit Inc., Daegu 43017, Republic of Korea; 4 Department of Molecular Biomedical Convergence, College of Biomedical Science, Kangwon National University, Chuncheon 24341, Republic of Korea; ah516@kangwon.ac.kr; 5 Department of Molecular Bioscience, College of Biomedical Science, Kangwon National University, Chuncheon 24341, Republic of Korea; 6 Division of Biomedical Convergence, College of Biomedical Science, Kangwon National University, Chuncheon 24341, Republic of Korea

**Keywords:** validamycin A, antibiotic fungicide, anti-seizure activity, zebrafish epilepsy model, electroencephalogram, angular velocity

## Abstract

Epilepsy is a prevalent neurological disorder characterized by recurrent seizures. Validamycin A (VA) is an antibiotic fungicide that inhibits trehalase activity and is widely used for crop protection in agriculture. In this study, we identified a novel function of VA as a potential anti-seizure medication in a zebrafish epilepsy model. Electroencephalogram (EEG) analysis demonstrated that VA reduced pentylenetetrazol (PTZ)-induced seizures in the brains of larval and adult zebrafish. Moreover, VA reduced PTZ-induced irregular movement in a behavioral assessment of adult zebrafish. The developmental toxicity test showed no observable anatomical alteration when the zebrafish larvae were treated with VA up to 10 µM within the effective range. The median lethal dose of VA in adult zebrafish was > 14,000 mg/kg. These results imply that VA does not demonstrate observable toxicity in zebrafish at concentrations effective for generating anti-seizure activity in the EEG and alleviating abnormal behavior in the PTZ-induced epileptic model. Furthermore, the effectiveness of VA was comparable to that of valproic acid. These results indicate that VA may have a potentially safer anti-seizure profile than valproic acid, thus offering promising prospects for its application in agriculture and medicine.

## 1. Introduction

Validamycin A (VA), a fungicidal agent, is primarily used in agriculture to mitigate rice plant blight, ear rot, and hairy blight [1,2]. VA was originally isolated from *Streptomyces hygroscopicus* var. It inhibits the growth of *Aspergillus flavus*, with a minimum inhibitory concentration of 1 μg/mL [3]. VA inhibits the activity of trehalase in *Rhizoctonia solani*, with a half-maximal inhibitory concentration of 72 μM [4], and acts as a reversible tyrosinase inhibitor, with an inhibitor constant (K_i_) of 5.893 mM [5].

Epilepsy is one of the most common neurological disorders. It affects 1–2% of the global population [6,7] and is a spectrum disorder with a variety of genetic and acquired causes [8]. It results from abnormally heightened or synchronous neuronal activity in the central nervous system. It is characterized by recurrent epileptic seizures, wherein uncontrolled electrical activity in the brain leads to variations in behavior, emotion, and consciousness [7,9]. Moreover, epileptic seizures can lead to several comorbidities, including anxiety, cognitive impairment, depression, and social dysfunction [7]. Epilepsy treatments are categorized into pharmacological and non-pharmacological approaches, such as surgery and nerve stimulation [10]. Despite the availability of more than 30 FDA-approved anti-seizure medications, one third of patients do not achieve adequate seizure control [11,12].

Zebrafish have been widely used as an experimental animal model to study brain function, neurological disorders, and drug toxicity. Electroencephalography (EEG) is a noninvasive diagnostic technique used for measuring cerebral neural activity. This method is predominantly used to diagnose epilepsy. In zebrafish, electrodes are placed near the telencephalon, a region in the forebrain and midbrain, facilitating real-time observation of brain activity via waveforms emanating from these areas. A system has been developed to simultaneously record EEG signals from multiple adult zebrafish under stable conditions for a specified duration, incorporating perfusion and recording systems [13]. Additionally, a system for simultaneously recording EEG signals in multiple zebrafish larval brains has also been developed [14]. Moreover, pentylenetetrazol (PTZ) treatment results in seizure-like ictal activity in EEG recordings of the zebrafish brain that resembles seizure activity in the brains of patients. Hence, zebrafish with PTZ-induced seizures are a useful model for evaluating potential antiepileptic medications [15].

Here, we aimed to measure EEG signals in the brains of larvae and adult zebrafish using PTZ-induced seizure models and assessed the anti-seizure activity of VA against that of valproic acid (VPA), a convergent medication. VA reduced PTZ-induced irregular velocity in a behavioral assessment. These results suggest that VA has the potential to be repurposed as an antiseizure medication.

## 2. Results

### 2.1. Assessment of the Anti-Seizure Efficacy of VA Using Zebrafish Larval Epilepsy Model

We investigated the anti-seizure efficacy of VA using a zebrafish larval epilepsy model in which seizure-like EEG activity was induced by PTZ. EEG signals were recorded to evaluate the potential reduction in seizure-like EEG activity in zebrafish larval brains.

**Figure 1:**
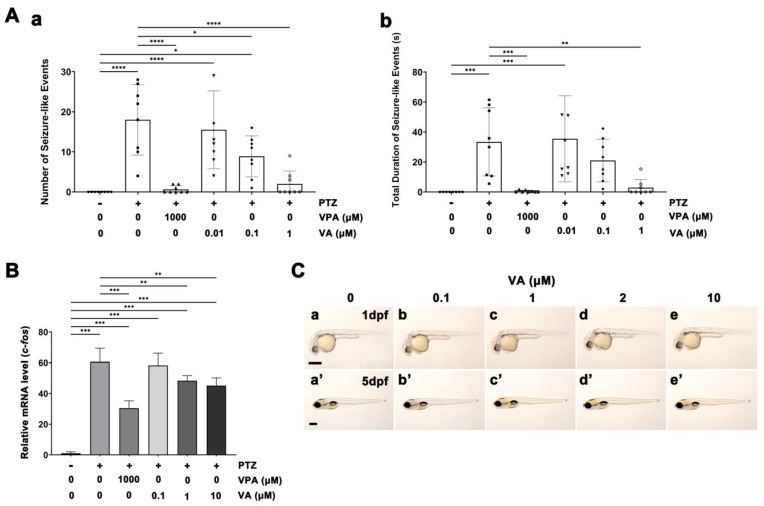
EEG analysis of anti-seizure efficacy of VA in the brains of PTZ-treated zebrafish larvae (**A**) EEG signals corresponding to PTZ-induced seizure-like activity were recorded in the brains of zebrafish larvae pre-treated with 0, 0.01, 0.1, and 1 μM VA or with 1000 μM VPA for comparison. The total seizure count (**a**) and cumulative duration of seizures (**b**) recorded during EEG analyses are shown. We examined 6 to 9 fish in each treatment group for EEG analysis. The results are expressed as the mean ± standard error of the mean (S.E.M.). A one-way analysis of variance (ANOVA) was conducted for statistical analysis, and the significance of the results was confirmed. * *p* < 0.05, ** *p* < 0.01, *** *p* < 0.001, **** *p* < 0.0001; (**B**) mRNA expression analysis of *c-fos* in 5 dpf zebrafish larvae using qRT-PCR. Data are presented as mean ± standard error of the mean (S.E.M.). Statistical significance was determined using Student’s *t*-test. ** *p* < 0.01, *** *p* < 0.001; (**C**) Lateral views of bright-field images of zebrafish embryos treated with VA at various concentrations that showed anti-seizure efficacy. Scale bar = 400 µm. The embryos at 1 dpf (**a**–**e**) and larvae at 5 dpf (**a’**–**e’**) showed no observable morphological abnormalities. The experiment was conducted with 24 to 54 fish in each treatment group.

The total number of PTZ-induced seizure events decreased in the VA-treated zebrafish larval brain in a dose-dependent manner compared to the number of seizures in the control without drug treatment (Figure 1A). Pre-treatment with 1 μM VA resulted in a statistically significant reduction in the total number of PTZ-induced seizure events and in seizure duration compared with the PTZ group (Figure 1A; Figure S1; Table S1). Hence, 1 μM VA showed anti-seizure efficacy comparable to that of 1000 μM VPA. Moreover, we measured *c-fos* mRNA expression in zebrafish larvae. PTZ-induced *c-fos* expression decreased in the VA-treated zebrafish larvae in a dose-dependent manner (Figure 1B). Pre-treatment with 1 μM and 10 μM VA significantly reduced PTZ-induced *c-fos* expression compared to the PTZ group (Figure 1B).

We examined whether VA demonstrated developmental toxicity in embryos exposed to VA during early development. Zebrafish embryos were treated with various concentrations of VA to evaluate anti-seizure efficacy. The embryos at one day post-fertilization (dpf) and larvae at five dpf showed no observable morphological abnormalities (Figure 1C).

### 2.2. Assessment of Anti-Seizure Efficacy of VA in the Adult Brain Using a Zebrafish Epilepsy Model

**Figure 2:**
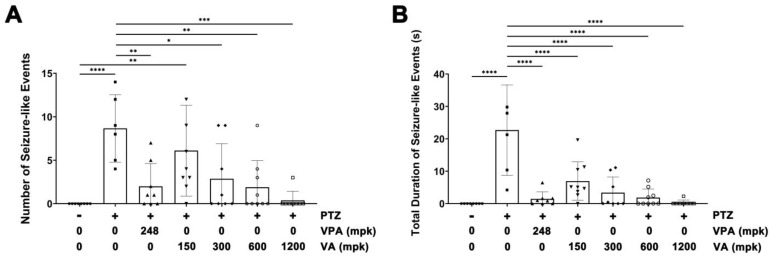
EEG analysis of the anti-seizure efficacy of VA in the brains of adult zebrafish administered PTZ. The collected data were represented graphically, depicting the total number of seizures (**A**) and cumulative duration (**B**). The experiment was conducted with 6 to 9 fish in each treatment group. The results are presented as mean ± standard error of the mean (S.E.M.). A one-way analysis of variance (ANOVA) was employed for the statistical analysis, and the significance of the findings was subsequently ascertained. * *p* < 0.05, ** *p* < 0.01, *** *p* < 0.001, **** *p* < 0.0001.

We evaluated the antiepileptic efficacy of VA against PTZ-induced seizures in adult zebrafish brains. VA was administered along with PTZ into the mouth of adult zebrafish, and EEG activity in the brain was monitored using noninvasive flat electrodes. The results revealed a dose-dependent decrease in overall seizure frequency, with increasing doses of VA relative to those observed in the PTZ control group (Figure 2A; Figure S2; Table S2). Simultaneously, the dose-dependent decline in the cumulative duration of seizures was substantial at a concentration of 300 mg/kg (mpk) compared with that in the PTZ group (Figure 2B; Figure S2; Table S2). Notably, the anti-seizure activity of VA was comparable to that of VPA. These results demonstrated the antiepileptic efficacy of VA in the adult zebrafish brain at druggable concentrations.

### 2.3. Assessment of the Efficacy of VA via Angular Velocity Analysis of Adult Zebrafish Movement

**Figure 3:**
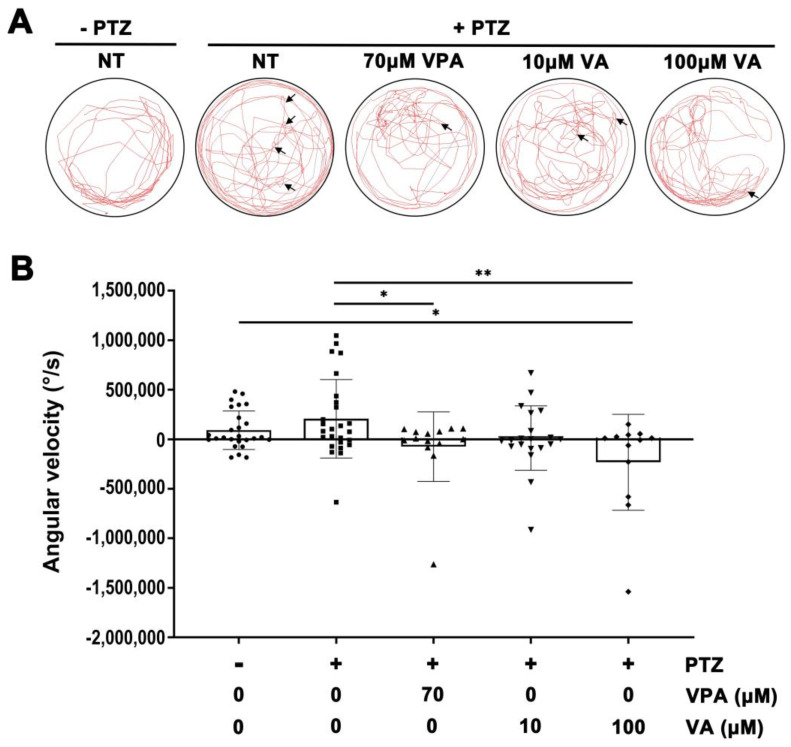
Analysis of validamycin A’s efficacy in mitigating locomotive changes in adult zebrafish via measurements of angular velocity. (**A**) The red lines and arrows indicate the locations of the zebrafish and abrupt changes in movement direction during a 1-min video recording in a 90 mm Petri dish. (**B**) Angular velocity was calculated. The sample size for each experimental group ranged from 12 to 26 fish. The collected data are presented as the mean ± standard error of the mean (S.E.M.). Student’s *t*-test was utilized for the statistical analysis to determine the significance of the observed differences. * *p* < 0.05, ** *p* < 0.01.

We assessed the effectiveness of VA in relieving PTZ-induced behavioral alterations in adult zebrafish in water. We measured the angular velocity of movement in adult zebrafish and examined the effect of the drug on irregular movement due to PTZ-induced seizures. Adult zebrafish were pretreated with VPA or VA in a 90 mm Petri dish for 30 min. Subsequently, PTZ was added to the water in the Petri dish to obtain a final concentration of 10 mM, and the locomotive activity of adult fish was monitored. The data demonstrated that VA reduced the PTZ-induced increment in angular velocity at a concentration of 100 μM, which was compatible with the inhibitory activity of 70 μM VPA (Figure 3). These results indicate that VA effectively suppresses seizure at this concentration, reflecting the antiepileptic effect of VA on the locomotive behavior of adult zebrafish.

### 2.4. Calculation of the Therapeutic Dosage of VA

**Figure 4:**
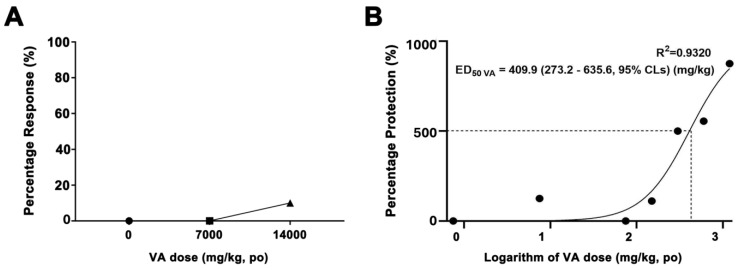
Quantification of the therapeutic dosage of validamycin A. (**A**) The half-lethal dose (LD50) was evaluated by determining the survival rate following oral administration of VA to zebrafish at doses of 0, 7000, and 14,000 mg/kg (mpk); (**B**) Half-effective dose (ED50) was determined after administering varying concentrations of VA—0, 0.75, 7.5, 75, 150, 300, 600, and 1200 mpk—alongside 220 mpk of PTZ to the fish. The ED50 was measured based on the presence or absence of epileptiform discharges. The therapeutic index was calculated by dividing the LD50 by the ED50. For VA, the therapeutic index was determined to be above 34.2, indicating a high safety margin for the drug. Each experimental group comprised 8–10 fish.

We determined the median lethal dose (LD50) and half-effective dose (ED50) of VA and established its therapeutic index (TI) in adult zebrafish. The TI of each drug was calculated as the ratio of its LD50 to its ED50. We orally administered doses of 0, 7000, and 14,000 mg/kg (mpk) to adult zebrafish in each treatment group and assessed the mortality over 96 h. Only a single fatality was recorded among the eight zebrafish at the 14,000 mpk dose, and all the others survived, indicating that the LD50 of VA exceeded 14,000 mpk (Figure 4A). Additionally, ED50 was ascertained by evaluating the presence or absence of epileptiform discharges in EEG signals of adult zebrafish administered VA at 0, 0.75, 7.5, 75, 150, 300, 600, and 1200 mpk along with 220 mpk PTZ. The ED50 was determined to be 409.9 mpk (Figure 4B). The TI of VA was determined to be > 34.2, indicating that VA has a favorable safety profile.

## 3. Discussion

The significance of a vast number of chemicals in modern science can be redefined in different fields by precision technology, which enables the evaluation of their functions from different perspectives. This enables their repurposing or the identification of previously unrecognized toxicity. VA is used as a pesticide. Here, we address the crucial challenges associated with determining the potential role in human health.

In this study, we used a zebrafish model with PTZ-induced seizures and simultaneously recorded the brain activity of multiple larval and adult zebrafish using multichannel EEG recordings, as previously described [13]. We demonstrated that VA reduced PTZ-induced seizures in the brains of both zebrafish larvae and zebrafish adults.

We examined the effect of VA on countering the behavioral changes in adult zebrafish induced by PTZ. We demonstrated that VA reduced PTZ-induced irregularities in angular velocity, indicating that the effects of antiepileptic medications can be evaluated by analyzing movement patterns in adult zebrafish. This suggests that the antiepileptic effect of VA is discernable through the locomotive behavior of adult zebrafish. Notably, behavioral changes associated with the effectiveness of VA were evident at 100 μM, whilst the effectiveness of VA observed in the EEG recordings depicting anti-seizure activity was statistically significant with effects evident at 300 mpk and above. The differences in drug-exposure systems led to variation in the units of drug concentrations. Micromolar units were used when larvae or adult fish were immersed in solutions containing the drugs, whereas mpk units were employed when drugs were administered to the mouths of the zebrafish via perfusion systems.

The primary goal of pharmacotherapy for patients with epilepsy is to control seizures while minimizing drug toxicity, thereby enhancing the patients’ quality of life [9,16]. First-generation antiepileptic drugs include VPA, benzodiazepine, phenobarbital, primidone, and carbamazepine, whereas second-generation medications include lamotrigine, levetiracetam, topiramate, zonisamide, and oxcarbazepine [16]. However, adverse effects are common, and approximately one-third of patients with epilepsy develop drug resistance. Drug-resistant epilepsy refers to seizures that remain uncontrolled despite the use of two or more antiepileptic medications. This resistance increases the risk of disability and premature mortality associated with seizures [7,16].

VPA is currently used as an antiepileptic medication; however, it demonstrates developmental toxicity during zebrafish embryogenesis [17]. Here, we present findings that the in vivo antiseizure activity of VA is comparable to that of VPA in a zebrafish model of PTZ-induced epilepsy. VA demonstrated remarkably lower toxicity during zebrafish embryogenesis at concentrations effective for anti-seizure activity. This finding suggests that VA is a promising candidate for therapeutic applications and has great potential for applications in both the agricultural and medical fields. Unraveling the mechanism underlying its effect is crucial for advancing its application.

## 4. Materials and Methods

### 4.1. Zebrafish Maintenance

Wild-type AB zebrafish aged 3–12 months were used in this study and maintained at 27 °C in a controlled environment under a 14-h light/10-h dark cycle. Embryos were obtained via natural spawning and subsequently cultured in the E3 medium (5 mM NaCl, 0.33 mM MgSO_4_, 0.33 mM CaCl_2_, and 0.17 mM KCl) in a Petri dish at 28.5 °C until they reached the developmental stages of interest. The animal procedures were approved by the Animal Use and Ethics Committee of Kangwon National University (Protocol KW- 171227-2) and performed in accordance with the National Law for Laboratory Animal Experimentation guidelines.

### 4.2. Electroencephalographic Evaluation of Brain Wave Signals in Zebrafish Larvae

Seizures were elicited in the larvae by administering PTZ, a well-known convulsant extensively used to induce seizure activity in animal models [18]. Individual zebrafish larvae at 6 days post-fertilization (dpf) were pre-treated with VA at 0, 0.01, 0.1, 1 μM in 1% DMSO/E3 medium in a 96-well plate for 18 h, with this treatment followed by a 15-min exposure to 15 mM PTZ. The pre-treatment with 1000 μM VPA, an antiepileptic medication, was used as a comparison. The larvae were then transferred into an EEG-recording chamber using an 18-gauge needle to ensure proper positioning, and invasive electrodes were placed on the larval brains. EEG signals were recorded for 10 min and processed using an MP36 device (Biopac Systems Inc., Goleta, CA, USA) at Zefit Inc. (Daegu, Republic of Korea). We examined eight larvae in each treatment group for EEG analysis.

The EEG data were recorded from PTZ-induced zebrafish epilepsy models, focusing on the characterization of seizure-like events. These events demonstrated distinct large-amplitude, polyphasic, ictal-like multi-spike waveforms with voltage deflections exceeding five times the baseline. The waveforms exhibited prolonged durations, each exceeding 300 milliseconds, ensuring comprehensive data collection for rigorous statistical analysis.

### 4.3. VA Treatment and Quantitative Reverse Transcription Polymerase Chain Reaction (qRT-PCR)

Zebrafish larvae at four dpf were pre-treated with VA at concentrations of 0, 0.1, 1, and 10 μM in 1% DMSO/E3 medium in a 24-well plate for 18 h; this treatment was followed by a 1-h exposure to 15 mM PTZ. A pre-treatment with 1000 μM VPA, an antiepileptic medication, was used as a comparison. Total RNA was extracted from 5 larvae using the RNeasy MinElute Cleanup Kit (74204, QIAGEN, Hilden, Germany). cDNA was synthesized using the PrimeScript 1st strand cDNA Synthesis Kit (6210A, Takara, Kusatsu, Japan) according to the manufacturer’s instructions. Quantitative PCR (qPCR) was performed on the QuantStudio 1 Real-Time PCR system (Thermo Fisher Scientific, Waltham, MA, USA) using SYBR Green with low ROX (RT500M, Enzynomics, Daejeon, Republic of Korea); β-actin was used as the control. Each sample was run in triplicate. Gene expression was normalized to the expression level of β-actin, and transcripts were quantified using the 2^−ΔΔCt^ method.

The following primers were used: *β-actin* forward (5′-GTG ATG GTT GGC ATG GGA CAG-3′) and reverse (5′-CCA GTT GGT CAC AAT ACC GTG C-3′); *c-fos* forward (5′-CAG CTC CAC CAC AGT GAA GA-3′) and reverse (5′-GCT CCA GGT CAG TGT TAG CC-3′).

### 4.4. VA Treatment and Bright-Field Imaging of Live Zebrafish Embryos

Twenty zebrafish embryos in each treatment group were exposed to various concentrations of VA in a 35-mm Petri dish from 5 h post-fertilization (hpf) (50% epiboly stage) and raised in an incubator at 28.5 °C. The morphology of the developing embryos was examined at 1 dpf and 5 dpf using bright-field imaging. For bright-field imaging, the embryos were hatched and anesthetized with 0.02% tricaine (Sigma-Aldrich Corp., St. Louis, MO, USA) in the E3 medium, then mounted with 3% methylcellulose (Sigma-Aldrich Corp., St. Louis, MO, USA). Bright-field images of the embryos were captured using an Olympus SZX16 fluorescent stereoscope (Olympus, Tokyo, Japan) equipped with an AxioCam GRC camera (Carl Zeiss, Overkochen, Germany). The captured images were processed using ZEN software (version 3.0; Carl Zeiss, Overkochen, Germany) as previously described [18]. All experiments were repeated at least thrice.

### 4.5. Recording of EEG Signals in Adult Zebrafish Brain

Adult zebrafish were submerged in 15 ppm eugenol (Sigma-Aldrich Corp., St. Louis, MO, USA) in water for 1 h as a pre-anesthetic treatment to reach stage-3 anesthesia. Once anesthetized, the fish were immobilized in a holding device, and eugenol (7.5 ppm) was administered orally to sustain anesthesia while the fish were outside the water. Noninvasive electrodes were attached to the heads of the adult fish, and EEG recordings were performed for 20 min following the simultaneous oral administration of either the comparative drug VPA or the drug candidate VA along with PTZ, precisely overlaying the regions of the telencephalon and midbrain. EEG signals were captured and processed using an MP36 device (Biopac Systems Inc., Goleta, CA, USA) at Zefit Inc (Daegu, Republic of Korea).

### 4.6. Assessment of Angular Velocity of Adult Zebrafish Movement

The angular velocity (speed of turning) represents the magnitude of change in the direction of movement, as calculated between two consecutive time samples. An increase in angular velocity corresponds to a significant increase in the bending of the entire zebrafish body, which reflects erratic movements [19]. Angular velocity serves as an indicator of the irregular movement associated with seizures [20]. Each adult zebrafish was acclimated in 2 L of system water for 2 h and pretreated with VPA or VA in a 90-mm Petri dish for 30 min. Subsequently, PTZ was added to obtain a final concentration of 10 mM. The free-swimming behavior of the adult fish was captured using a SONY HDR-CX560 video camera (SONY, Tokyo, Japan), and the footage was analyzed for motility using the EthoVision XT 14.0 software (Noldus, Wageningen, The Netherlands). The angular velocity, indicative of motility in adult zebrafish, was quantified following pre-treatment with two concentrations of VA, specifically, 10 and 100 μM. Additionally, a group was treated with 70 μM VPA and 10 mM PTZ as a positive control. The sample size of each experimental group ranged from 12 to 26 fish.

### 4.7. Measurement of the Therapeutic Dosage of VA

We determined the LD50 and ED50 of VA and established its TI in adult zebrafish. The TI signifies the range within which a drug is effective without causing adverse effects, and a drug is generally deemed safe if its TI exceeds a value of 10. The TI of each drug was calculated as the ratio of its LD50 to its ED50. We orally administered doses of 0, 7000, and 14,000 mpk to adult fish and assessed the mortality over 96 h. Additionally, ED50 was ascertained by evaluating the presence or absence of epileptiform discharges through EEG signal analysis of adult zebrafish administered VA at 0, 0.75, 7.5, 75, 150, 300, 600, and 1200 mpk along with 220 mpk PTZ.

### 4.8. Statistical Analysis

All statistical analyses of the experimental data were performed using the SPSS version 24.0. (IBM Corp., Armonk, NY, USA) and Graph-Pad Prism 7.0 software (GraphPad Software Inc., La Jolla, CA, USA). The data are presented as quantitative values and are expressed as mean ± standard deviation. Significant differences between the control and treatment groups were determined using one-way analysis of variance and Student’s *t*-test. To determine statistical significance, the significance levels were set as follows: * *p* < 0.05, ** *p* < 0.01, *** *p* < 0.001.

## 5. Conclusions

We identified the anti-seizure activity of VA by screening various pesticides for neurodevelopmental toxicity during zebrafish embryogenesis. According to the present data, VA seems to be potentially safer than VPA for use in pregnant women as an anti-seizure medication with regard to teratogenic toxicity during fetal development. The unexpected findings of this study offer a perspective on VA applications in agriculture and medicine. Further studies on the molecular targets and cellular pathways affected by this potent compound are required before VA can be repurposed from an antibiotic to an epilepsy medication.

## Data Availability

Data are contained within the article and Supplementary Materials.

## Supplementary Materials

The following supporting information can be downloaded at: https://www.mdpi.com/article/10.3390/molecules29112572/s1, Figure S1: Representative EEG recordings in the brains of 6-day post-fertilization zebrafish larvae; Figure S2: Representative real-time EEG recordings in the brains of adult zebrafish; Table S1: Raw data sets in statistics for number of seizure-like events and Total duration of seizure-like events in Figure 1A; Table S2: Raw data sets in statistics for number of seizure-like events and total duration of seizure-like events in Figure 2.
