# Supplementary material for: Assessment of the Novel Anti-Seizure Potential of Validamycin A Using Zebrafish Epilepsy Model
Source: Molecules. 2024 May 30;29(11):2572. doi: 10.3390/molecules29112572 (PMC11173475; doi:10.3390/molecules29112572)
Supplement: Supplementary file 1 [file molecules-29-02572-s001.zip › molecules-2983132-supplementary.pdf]

molecules

Assessment of the Novel Anti-Seizure Potential of Validamycin A Using Zebrafish Epilepsy Model

Eunhye Lee [1], Amit Banik [2], Ki-Baek Lee [3], Seung Min Sim [1], Ah Hyun Kil [4], Byung Joon Hwang [4,5,*] and Yun Kee [4,6,*]

[1] Department of Biomedical Science, College of Biomedical Science, Kangwon National University, Chuncheon 24341, Republic of Korea
[2] Interdisciplinary Graduate Program in Environmental and Biomedical Convergence, College of Biomedical Science, Kangwon National University, Chuncheon 24341, Republic of Korea
[3] Zefit Inc., Daegu 43017, Republic of Korea
[4] Department of Molecular Biomedical Convergence, College of Biomedical Science, Kangwon National University, Chuncheon 24341, Republic of Korea
[5] Department of Molecular Bioscience, College of Biomedical Science, Kangwon National University, Chuncheon 24341, Republic of Korea
[6] Division of Biomedical Convergence, College of Biomedical Science, Kangwon National University, Chuncheon 24341, Republic of Korea
* Correspondence: bjhwang@kangwon.ac.kr (B.J.H.); yunkee@kangwon.ac.kr (Y.K.)

Supplementary Materials:

S1. Representative electroencephalogram (EEG) recordings in the brains of zebrafish larvae

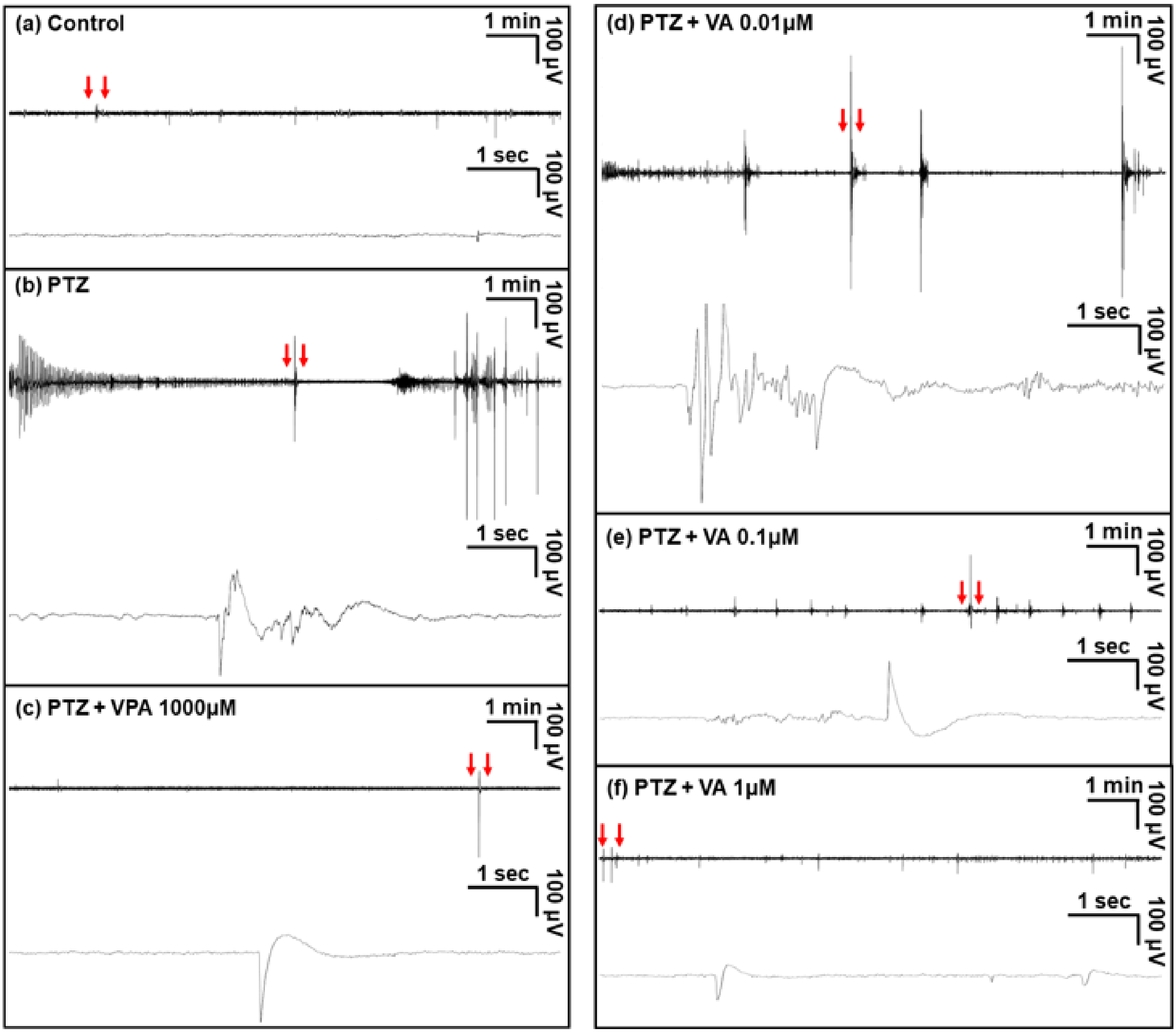

Figure S1. Representative EEG recordings in the brains of 6-day post-fertilization zebrafish larvae.
EEG signals were recorded in the brain of zebrafish larvae with no treatment as control (a) or with 15 mM pentylenetetrazol (PTZ) treatment (b), following pre-treatment with 1000 μM valproic acid (VPA) (c) or 0.01 μM (d), 0.1 μM (e), and 1 μM (f) of validamycin A (VA). Red arrows represent the magnified viewpoints of ictal signals.

Table S1. Raw data sets in statistics for number of seizure-like events and Total duration of seizure-like events in Figure 1A.

The EEG data were recorded from PTZ-induced zebrafish epilepsy models, focusing on the characterization of seizure-like events. These events demonstrated distinct large amplitude, polyphasic, ictal-like multi-spike waveforms, with voltage deflections exceeding five times the baseline. The waveforms exhibited prolonged durations, each exceeding 300 milliseconds, ensuring comprehensive data collection for rigorous statistical analysis.

The PTZ-induced seizure-like activity were shown as total seizure count (a) and total duration of seizures (b) in Figure 1A. We examined eight fish in each treatment group for EEG analysis. The results are expressed as the mean ± standard error of the mean (S.E.M.). All data were analyzed by one- way ANOVA. Statistical significance was accepted for $p < 0.05$, with indications of *p<0.05, **p<0.01, ***p<0.001, ****p<0.0001.

(a) Number of seizure-like events

Table S1-1. ***1st One-way ANOVA analysis***

(*NC, negative control; ns, nonsignificant)

Dunnett's multiple comparisons test	Mean Diff.	95.00% CI of diff.	Below threshold?	Summary	Adjusted P Value
NC vs. PTZ	-18.00	-25.72 to -10.28	Yes	****	<0.0001
NC vs. VPA 1000μM	-0.6250	-8.349 to 7.099	No	ns	0.9997
NC vs. VA 0.01μM	-15.50	-23.22 to -7.776	Yes	****	<0.0001
NC vs. VA 0.1μM	-8.875	-16.60 to -1.151	Yes	*	0.0191
NC vs. VA 1μM	-2.000	-9.724 to 5.724	No	ns	0.9395

Table S1-2. ***2nd One-way ANOVA analysis***

Dunnett's multiple comparisons test	Mean Diff.	95.00% CI of diff.	Below threshold?	Summary	Adjusted P Value
PTZ vs. NC	18.00	10.28 to 25.72	Yes	****	<0.0001
PTZ vs. VPA 1000μM	17.38	9.651 to 25.10	Yes	****	<0.0001
PTZ vs. VA 0.01μM	2.500	-5.224 to 10.22	No	ns	0.8656
PTZ vs. VA 0.1μM	9.125	1.401 to 16.85	Yes	*	0.0153
PTZ vs. VA 1μM	16.00	8.276 to 23.72	Yes	****	<0.0001

(b) Total duration of seizure-like events (s)

Table S1-3. *1st One-way ANOVA analysis*

Dunnett's multiple comparisons test	Mean Diff.	95.00% CI of diff.	Below threshold?	Summary	Adjusted P Value
NC vs. PTZ	-33.38	-54.52 to -12.24	Yes	***	0.0008
NC vs. VPA 1000μM	-0.4644	-21.61 to 20.68	No	ns	>0.9999
NC vs. VA 0.01μM	-35.45	-56.59 to -14.31	Yes	***	0.0004
NC vs. VA 0.1μM	-20.98	-42.13 to 0.1588	No	ns	0.0523
NC vs. VA 1μM	-2.923	-24.06 to 18.22	No	ns	0.9959

Table S1-4. *2nd One-way ANOVA analysis*

Dunnett's multiple comparisons test	Mean Diff.	95.00% CI of diff.	Below threshold?	Summary	Adjusted P Value
PTZ vs. NC	33.38	12.24 to 54.52	Yes	***	0.0008
PTZ vs. VPA 1000μM	32.92	11.78 to 54.06	Yes	***	0.0009
PTZ vs. VA 0.01μM	-2.071	-23.21 to 19.07	No	ns	0.9992
PTZ vs. VA 0.1μM	12.40	-8.744 to 33.54	No	ns	0.4117
PTZ vs. VA 1μM	30.46	9.317 to 51.60	Yes	**	0.0023

S2. EEG recordings in the brains of adult zebrafish

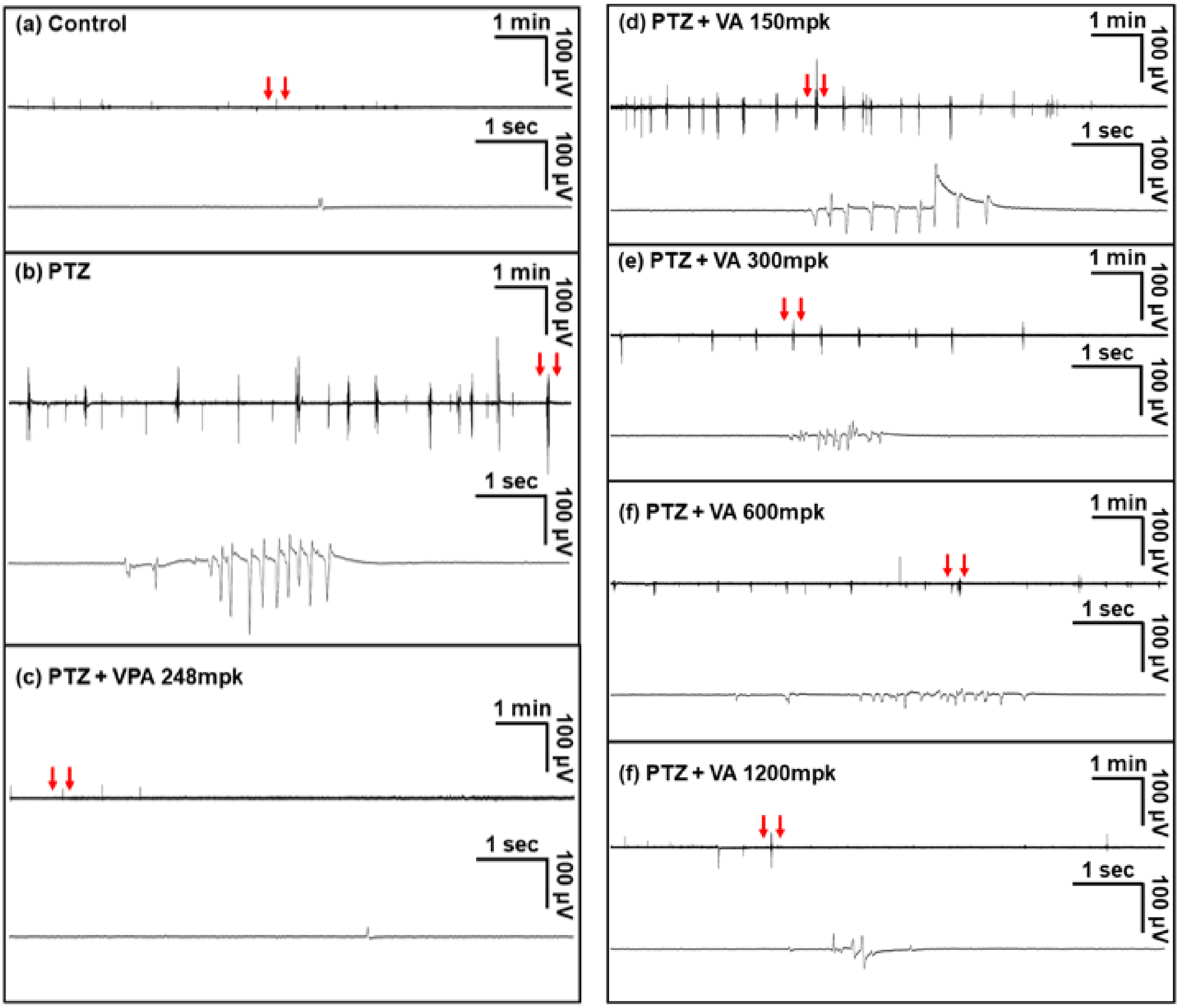

Figure S2. Representative real-time EEG recordings in the brains of adult zebrafish.

EEG signals were recorded in the brain of adult zebrafish with no treatment as control (a) or with 15 mM pentylenetetrazol (PTZ) treatment (b), following pre-treatment with 1000 μM valproic acid (VPA) (c) or 0.01 μM (d), 0.1 μM (e), and 1 μM (f) of validamycin A (VA). Red arrows represent the magnified viewpoints of ictal signals.

Table S2. Raw data sets in statistics for number of seizure-like events and total duration of seizure-like events in Figure 2.

The EEG analyses of PTZ-induced seizure-like activity were shown as total seizure count (a) and total duration of seizures (b) in Figure 2. We examined six to eight fish in each treatment group for EEG analysis. The results are expressed as the mean ± standard error of the mean (S.E.M.). All data were analyzed by one- way ANOVA. Statistical significance was accepted for $p < 0.05$, with indications of *p<0.05, **p<0.01, ***p<0.001, ****p<0.0001.

(a) Number of Seizure-like Events

Table S2-1. *1st One-way ANOVA analysis*

(*NC, negative control; ns, nonsignificant)

Dunnett's multiple comparisons test	Mean Diff.	95.00% CI of diff.	Below threshold?	Summary	Adjusted P Value
NC vs. PTZ	-8.667	-13.43 to -3.908	Yes	****	<0.0001
NC vs. VPA 248 mpk	-2.000	-6.406 to 2.406	No	ns	0.6823
NC vs. VA 150 mpk	-6.111	-10.39 to -1.829	Yes	**	0.0022
NC vs. VA 300 mpk	-2.875	-7.281 to 1.531	No	ns	0.3317
NC vs. VA 600 mpk	-1.889	-6.171 to 2.393	No	ns	0.7064
NC vs. VA 1200 mpk	-0.3750	-4.781 to 4.031	No	ns	0.9999

Table S2-2. 2nd One-way ANOVA analysis

Dunnett's multiple comparisons test	Mean Diff.	95.00% CI of diff.	Below threshold?	Summary	Adjusted P Value
PTZ vs. NC	8.667	3.965 to 13.37	Yes	****	<0.0001
PTZ vs. VPA 248 mpk	6.667	1.965 to 11.37	Yes	**	0.0026
PTZ vs. VA 150 mpk	2.556	-2.033 to 7.144	No	ns	0.4650
PTZ vs. VA 300 mpk	5.792	1.090 to 10.49	Yes	*	0.0105
PTZ vs. VA 600 mpk	6.778	2.190 to 11.37	Yes	**	0.0016
PTZ vs. VA 1200 mpk	8.292	3.590 to 12.99	Yes	***	0.0001

(b) Total Duration of Seizure-like Events (s)

Table S2-3. 1st One-way ANOVA analysis

Dunnett's multiple comparisons test	Mean Diff.	95.00% CI of diff.	Below threshold?	Summary	Adjusted P Value
NC vs. PTZ	-22.71	-30.69 to -14.72	Yes	****	<0.0001
NC vs. VPA 248 mpk	-1.446	-8.838 to 5.947	No	ns	0.9894
NC vs. VA 150 mpk	-6.960	-14.14 to 0.2245	No	ns	0.0609
NC vs. VA 300 mpk	-3.389	-10.78 to 4.003	No	ns	0.6738
NC vs. VA 600 mpk	-1.858	-9.042 to 5.327	No	ns	0.9596
NC vs. VA 1200 mpk	-0.2821	-7.675 to 7.110	No	ns	>0.9999

Table S2-4. 2nd One-way ANOVA analysis

Dunnett's multiple comparisons test	Mean Diff.	95.00% CI of diff.	Below threshold?	Summary	Adjusted P Value
PTZ vs. NC	22.71	14.82 to 30.60	Yes	****	<0.0001
PTZ vs. VPA 248 mpk	21.26	13.38 to 29.15	Yes	****	<0.0001
PTZ vs. VA 150 mpk	15.75	8.051 to 23.45	Yes	****	<0.0001
PTZ vs. VA 300 mpk	19.32	11.43 to 27.21	Yes	****	<0.0001
PTZ vs. VA 600 mpk	20.85	13.15 to 28.55	Yes	****	<0.0001
PTZ vs. VA 1200 mpk	22.43	14.54 to 30.32	Yes	****	<0.0001
